# Tick-Borne Encephalitis Serological Survey of Students from University of Liège

**DOI:** 10.3390/v17091235

**Published:** 2025-09-11

**Authors:** Claude Saegerman, Constance Wielick, Gilles Darcis, Nicole Maréchal, Fabrice Bureau, Laurent Gillet, Anne-Françoise Donneau, Kevin K. Ariën, Marjan Van Esbroeck

**Affiliations:** 1 Research Unit of Epidemiology and Risk Analysis Applied to Veterinary Science (UREAR-ULg), Fundamental and Applied Research for Animals & Health (FARAH) Center, Faculty of Veterinary Medicine, University of Liège, 4000 Liège, Belgium; cwielick@uliege.be; 2 Infectious Diseases Department, Liège University Hospital, 4000 Liège, Belgium; gdarcis@chuliege.be (G.D.); nmarechal@chuliege.be (N.M.); 3 Laboratory of Cellular and Molecular Immunology, GIGA Institute, University of Liège, 4000 Liège, Belgium; fabrice.bureau@uliege.be; 4 Laboratory of Immunology-Vaccinology, FARAH, University of Liège, 4000 Liège, Belgium; l.gillet@uliege.be; 5 Research Unit Public Health: From Biostatistics to Health Promotion, University of Liège, 4000 Liège, Belgium; afdonneau@uliege.be; 6 Department of Biomedical Sciences, Institute of Tropical Medicine, 2000 Antwerp, Belgium; karien@itg.be; 7 Virology Unit, Department of Biomedical Sciences, Institute of Tropical Medicine Antwerp, 2000 Antwerp, Belgium; 8 National Reference Center for Arboviruses, Department of Clinical Sciences, Institute of Tropical Medicine, 2000 Antwerp, Belgium; mvesbroeck@itg.be

**Keywords:** tick-borne encephalitis virus, tick-borne disease, tick, human, Belgium, emerging disease

## Abstract

Background: Zoonotic risks in exposed students are poorly documented in Belgium. According to the literature, even though human tick-borne encephalitis (TBE) infection risk has increased significantly in southern Belgium, no previous human serological survey has demonstrated specific antibodies directed at TBE virus. Methods and principal findings: The aim of this paper was to perform a representative serological survey on sera involving students at the University of Liege, in the southern part of Belgium, to discover possible exposure to TBEV. A total of 207 sera samples were randomly chosen and analyzed using ELISA IgM (with 1 positive student out of 207) and ELISA IgG (with 10 positive students out of 207), subsequent serial immunofluorescence antibody testing (IFAT) IgG (with 8 positive students out of 10 positive in ELISA IgG) and serial IFAT IgM (with 1 negative student out of 1 positive in ELISA IgM), and confirmatory tests, i.e., 50% and 90% plaque reduction neutralization tests (PRNTs) (1 strongly positive student out of 8 positive in IFAT). Conclusions and significance: The exposure of students from the southern part of Belgium (area with increasing risk) to TBEV was assessed for the first time. Antibodies against TBEV could only be demonstrated in 1 out of 207 students. This finding contributes to better decision-making in public health and prevention and management of tick-borne diseases in the context of climate change. Awareness among all students should be prioritized, with prevention measures against tick bites, particularly during forest and recreational activities contributing to risk, to maintain the current low seroprevalence levels.

## 1. Introduction

Zoonoses in veterinary students covering 55 years in 24 counties were reviewed [1]. It appeared that a limited number of studies were dedicated to this matter and most of these studies were related to case reports. Apart from a survey on factors determining the implementation of measures aimed at preventing zoonotic diseases in veterinary practices [2], no studies were performed in Belgium. The systematic review of Sánchez et al. [1] concluded that more studies are needed to understand the zoonotic risk of exposed veterinary students. In line with this recommendation, we decided to design a serological survey on archived sera from students included in the SARSSURV cohort from the University of Liege [3] and who gave informed consent for their sera to be used in testing for different zoonotic agents. This survey targeted tick-borne encephalitis (TBE).

Tick-borne encephalitis virus (TBEV) is a spherical, enveloped, positive-sense, single-stranded RNA virus and member of the genus *Orthoflavivirus,* in the family *Flaviviridae* [4], and is the causative agent of tick-borne encephalitis in humans. The RNA genome of TBEV consists of a single open reading frame encoding a polyprotein, which is subsequently cleaved into 10 viral proteins. Among these, three structural proteins, the capsid, pre-membrane, and envelope, assemble to form the virion. The capsid protein plays a central role in virus entry by mediating receptor binding and membrane fusion, and it represents the main target for neutralizing antibodies [5].

Tick-borne encephalitis (TBE) stands among the most serious vector-borne infections in Europe and many parts of Asia, caused by TBEV [4]. After infection with the European subtype of TBEV, the illness typically follows a biphasic course. The first phase, occurring about a week after exposure, is the viremic stage—marked by non-specific flu-like symptoms such as fever, fatigue, headache, muscle aches, and nausea—lasting several days (commonly around 5 days) [6]. If the infection progresses, the second phase begins, involving neurological manifestations such as meningitis or encephalitis. Persistent neurological sequelae have been observed in a minority of patients with severe disease, most commonly manifesting as paresis, though seizures and headaches have also been reported. The mortality rate is estimated at 1–2% [7,8]. In children, the disease typically follows a milder clinical course, with neurological complications occurring less frequently than in adults [9].

IgM antibodies are typically detectable at the onset of central nervous system (CNS) symptoms or within a few days thereafter and generally remain detectable for approximately six weeks following symptom onset [10]. IgG antibodies against TBEV are usually present at the onset of CNS symptoms, peak around six weeks post-onset, and can persist for several years. Antibody titers following natural infection are generally significantly higher than those induced by vaccination [11].

Diagnosing Orthoflavivirus infections using serology is difficult because antibodies often cross-react among closely related viruses. Cross-reactivity within the *Orthoflavivirus* genus has now been well documented (e.g., [12,13,14,15,16]). To address this, several strategies have been developed to enhance the specificity of serological tests [17]. To assess this, samples testing positive for IgG antibodies against dengue, yellow fever, hepatitis C, Japanese encephalitis, West Nile, and Zika viruses were analyzed using ELISA 2.0 IgG. The findings suggest that cross-reactivity is unlikely with antibodies against yellow fever and hepatitis C viruses, whereas it appears more probable with antibodies directed against dengue, Japanese encephalitis, West Nile, and Zika viruses. Diagnostic tests for anti-TBEV IgM are usually more specific than anti-TBEV IgG tests in terms of cross-reactivity with other Orthoflavivirus members [10].

In Europe, two vaccines against TBE are currently available: FSME–IMMUN^®^ (Pfizer Europe, Brussels, Belgium) and Encepur^®^ (Bavarian Nordic, Hellerup, Denmark) [18]. Both formulations contain inactivated viruses of the European TBEV subtype. FSME–IMMUN^®^ has additionally been shown to elicit cross-reactive immune responses against other subtypes [19,20]. The approved vaccination protocol consists of a three-dose primary immunization, followed by a booster dose within three years after the initial series and subsequent boosters every three to five years depending on age [21]. In endemic regions, national immunization advisory groups (NITAGs) generally recommend vaccination for individuals aged one year and older living in high-risk areas, as well as for persons with occupational exposure such as forest workers [22].

In Belgium, TBE surveillance is based on laboratory confirmation in suspected human cases [23,24], TBE seroprevalence monitoring in animals [25,26,27,28,29], and PCR testing of ticks collected from humans, from animals, and by flagging [29,30,31]. In humans, the number of TBE cases diagnosed in Belgium remains limited, and most of these are infections contracted while traveling to countries where the risk of the disease is known (e.g., [24]). The first three confirmed autochthonous cases were diagnosed in the summer 2020 [32]. Already in 2018, two possible/probable cases of infection in Belgium were reported, but the patients had also traveled during the incubation period. In animals, several studies carried out on different populations of domestic animals (cattle, sheep, dogs) and wild animals (deer, roe deer, and wild boar) [25,26,27,28,29,33] have shown that TBEV has been circulating in Belgium for several years now. Using a citizen science approach based on an existing notification tool for tick bites, 1599 and 928 ticks removed from humans, 99% of which belonged to *Ixodes ricinus*, were collected across Belgium in 2017 and 2021, respectively [23,30,31]. In addition, 1983 ticks were collected in areas showing the highest TBEV seroprevalence in Belgian wild boars [29]. No TBEV-RNA-positive ticks were detected in these studies. In 2019, a seroprevalence study in Flanders among 195 forestry workers exposed to tick bites during professional activities, of which 85% had more than 10 years of exposure and 42% reported at least one tick bite per month during the tick season, revealed that none had antibodies showing evidence of infection [34]. In May 2024, TBEV was detected for the first time in the province of Liège, in questing *Ixodes ricinus* ticks collected by flagging at the exact location where a patient had reportedly been bitten by a tick before developing TBE in Belgium in 2020 [35].

Recent expert knowledge elicitation identified nine important drivers of increase in human TBE incidence in Europe (in decreasing order): (i) changes in human behavior/activities; (ii) changes in eating habits or consumer demand (in some cases, the origin of TBE is the consumption of raw milk products) [7]; (iii) alterations in landscape structure; (iv) the role of humidity in pathogen persistence and transmission; (v) challenges in managing reservoir hosts and/or vectors; (vi) the impact of temperature on viral viability and spread; (vii) the diversity and number of wildlife populations acting as reservoirs or amplification hosts; (viii) the rise in indigenous wild mammal populations; (ix) the diversity and geographical distribution of tick vector species [36].

Several drivers were also found according to a recent literature review, i.e., factors linked to vegetation cover, climate, and the presence of tick hosts [37]. Another multi-scale study also suggests that high habitat richness reduces the risk of TBE in Europe [38].

Through multi-scale modeling of annual fluctuations in human TBE case risk across Europe, findings revealed a pronounced upward trajectory in infection probability within northwestern and southwestern regions. High-risk zones are consistently associated with the presence of principal tick-host species, robust human recreational activity in forested environments, sharp declines in late-summer temperatures, and elevated levels of annual precipitation [39].

Areas with the most significant increases in human TBE infection risk are concentrated in western Germany, southern Belgium, eastern France, and Switzerland [39].

The aim of this study was to perform a TBE serological survey using recently archived sera from students and newly recruited students at the University of Liege that study in the southern part of Belgium, a newly identified area at risk, to assess possible exposure to TBEV.

## 2. Materials and Methods

### 2.1. Ethics Committee and Informed Consent

This study received approval from the University Hospital of Liège Ethics Committee (reference number 2024/61, dated 29 May 2024). Informed consent was collected from all participants. Those involved in the previous SARSSURV study (protocol code 2021/96; 26 March 2021) had agreed to the use of their remaining serum samples for future research [3], while the newly recruited students consented to having their samples tested for multiple zoonotic pathogens. Compliance with data protection regulations was approved by the official data protection officer of the University of Liège.

### 2.2. Location of This Study

This study was conducted at the University of Liège. The University of Liege represents 26,641 students distributed in 11 faculties (59% female students and 41% male students; 24% foreign students and 130 nationalities represented; 51% bachelor’s, 38% master’s, and 10% doctorate and doctoral training students). Liège is located in the south-east of Belgium, close to western Germany.

### 2.3. Sampling Collection and Size

To be able to detect TBEV, we calculated the sample size (*N* = 195) using WinEpi (http://www.winepi.net/uk/ (accessed on 15 August 2025)) [40] considering a 95% confidence level, a population size of 626 students (sampling frame, i.e., available cohort inside the SARSSURV study) and an expected seroprevalence of 1.35% [40]. The expected prevalence was based on the middle of the range observed in general population, between 1 and 1.7% according to a recent review and meta-analysis that included small percentage of vaccinated individuals [41].

Serum samples were collected from the serum bank of a university-population-based prospective cohort study on SARS-CoV-2 infection and immunity (SARSSURV-ULiège) [3] (*N* = 126). The samplings were performed from 25 June 2021 to 22 December 2022 (median = 29 June 2022: inter-quartiles: 25 February 2022–17 October 2022). To reach a sufficient sample size of veterinary students, additional veterinary students were recruited from 22 October 2024 to 18 February 25 (*N* = 81). A total of 207 serum samples were tested.

### 2.4. Diagnostic Tests

All tests, except NS1-IgG ELISA, were performed at the National Reference Center (NRC) for Arboviruses, hosted by the Institute of Tropical Medicine, Antwerp, Belgium.

#### 2.4.1. ELISA Test

All 207 samples were screened by two commercial enzyme-linked immunosorbent assays (ELISAs) to detect IgM and IgG directed against TBEV and were used in parallel (anti-TBE Virus ELISA 2.0 (IgG) and Anti-TBE Virus ELISA (IgM); EUROIMMUN, Lübeck, Germany). Both ELISAs were performed according to the manufacturer’s instructions (EI 2661-9601-2 G for IgG antibodies and EI 2661-9601 M for IgM antibodies).

#### 2.4.2. NS1-IgG ELISA

Differentiation between vaccine-induced and infection-induced antibodies was performed using NS1-IgG ELISA (three subtypes, TBEV-EU, TBEV-Sib, and TBEV-FE) at the National Consulting Laboratory for TBE (Gerhard Dobler, Munich, Germany) [42]. This in-house assay was validated for diagnostic purposes in accordance with EU norm 16189.

#### 2.4.3. IFA Test

The IFA test is known to be more specific than ELISA and offers a good alternative method in case of questionable or borderline results in ELISA [43]. The anti-TBE virus immune fluorescence assay (IFA) (EUROIMMUN AG, https://www.euroimmun.com (accessed on 15 August 2025)) was used to detect TBEV IgG or IgM antibodies in serum from ELISA-positive students [32]. An IFA that detects IgG antibodies against TBEV performs specifically well if there is only a TBEV infection or vaccination in the medical history; in contrast, diagnosis of individuals with a history of infection or vaccination by a Orthoflavivirus other than TBEV can be difficult due to cross-reacting antibodies [10].

#### 2.4.4. Neutralization Test

Six serial dilutions of heat-inactivated serum (1/10-1/320 in DMEM) were incubated for 1 h at 37 °C/7% CO_2_ with a pre-titrated amount of 5 plaque-forming units of TBEV (Hypr strain). Sample–virus mixtures were added to previously seeded A549 cells (25,000/well on day-1) on a 96-well plate and incubated for 2 h (at 37 °C/7% CO_2_); whereafter, a 1% carboxymethyl cellulose overlay was added. After a four-day incubation period (at 37 °C/7% CO_2_), the supernatant was removed, and the cells were treated with 3.7% formaldehyde (30 min) and stained with a 0.1% Naphthol Blue Black (NBB) solution (30 min). After removal of the NBB, the cells were rinsed with tap water, the plates were dried, and the wells were scored as infected (presence of plaques) or neutralized (no plaques). The Reed–Muench method was used to calculate the neutralizing antibody titers that reduced the number of infected wells by 50% (PRNT50) and 90% (PRNT90) [24,44].

### 2.5. Epidemiological Investigation

The anonymity of the survey limited this investigation. However, a medical doctor contacted each student who tested positive. With their agreement, some contextual epidemiological information of interest were collected (lifestyle, eventual symptoms, vaccination history, and risk exposure) [45].

### 2.6. Statistical Analysis

The representativeness of the samples relative to the sampling frame (SARSSURV study) was tested by comparing the faculty origin using a Pearson correlation test [46]. If the Pearson coefficient was close to 1 and the *p*-value was less than 0.05, the representativeness was deemed acceptable.

The 95% CI of the prevalence was estimated using a Binomial Exact distribution (Stata SE 14.2, College Station, TX, USA).

The effect of the faculty origin of students on the seropositivity in ELISA IgG was tested using Fisher’s exact test [47].

## 3. Results

Considering the sampling frame of students (archived sera from the SARSSURS study), the Pearson coefficient correlation between the number of tested students and non-tested students as a function of the origin of the faculty was calculated as 0.984 (*p*-value < 0.0001). Consequently, student participant representativeness was considered acceptable.

Among the 207 tested participants, 11 were positive in ELISA, i.e., one was positive for ELISA IgM and 10 were positive for ELISA IgG. We did not observe any relationship between participants tested positive by ELISA (IgG or IgM) and the faculty of origin (Fisher’s exact test; *p*-value = 0.34) (Table 1). The two ELISA IgG-positive samples with the lowest ratios and the one ELISA IgM-positive sample were not confirmed by the IFA. Using PRNT 50 and PRNT 90, only one participant was confirmed positive for TBE infection with high anti-TBEV IgG titers, 225 and 106, respectively. The true prevalence (1/207) was estimated as 0.48% (95% CI: 0.012–2.66).

Epidemiological investigations related to the 11 participants seropositive in ELISA IgG or IgM, are depicted in Table 2. All eleven participants were contacted by a medical doctor but only seven gave contextual epidemiological information. For the seven participants that gave information, four were vaccinated for Orthoflavivirus (one for TBE and three for yellow fever), and one of them experienced dengue clinical presentation and the three others originated form Cameroon where vaccination for yellow fever is mandatory (https://www.wanda.be/en/landen/cameroon/ (accessed on 15 August 2025)).

In the absence of epidemiological information regarding the single IFA-confirmed positive sample, and in order to differentiate between vaccine-induced and infection-induced antibodies, the samples were further tested for IgG antibodies targeting non-structural protein 1 (NS1), using NS1-IgG ELISA against all three TBEV subtypes (i.e., TBEV-EU, TBEV-Sib, TBEV-FE). The results were negative for all three subtypes, indicating no evidence of a past TBEV infection and suggesting that the antibodies were most likely vaccine-induced.

Among the newly recruited students who completed the associated epidemiological survey (n = 81), 45 did not visit another country in the three months prior. Of the remaining 36 participants, the most frequently visited countries were France (23 occurrences), followed by Canada (3 occurrences), Luxembourg, Switzerland, the USA, and China (2 occurrences each) and Germany, Spain, Greece, the United Kingdom, Brazil, New Zealand, and Uzbekistan (1 occurrence each). Note that a single participant may have visited multiple countries within the three-month period.

## 4. Discussion

It should be emphasized and underlined that this study concerns a sample of the population (students from an area at risk) and does not represent the general Belgian population.

The exposure of students from the southern part of Belgium (area with increasing risk) [35,39] to TBEV was assessed for the first time. Antibodies against TBEV could only be demonstrated in 1 out of 207 students (0.48%) based on TBE PRNT. Based on the negative result of NS1 ELISA, we concluded that these antibodies were the result of vaccination.

Although the objective of this study was not to determine the cause of any false positive reactions to TBEV, one positive IgM serum was observed with the ELISA test but not confirmed with IFAT, which is more specific than the ELISA test. Cross-reaction with other Flaviviridae, especially of the same serocomplex, like Powassan virus (POWV), Omsk hemorrhagic fever virus (OHFV), Kyasanur Forest disease virus (KFDV), and louping-ill virus (LIV), is possible [48]. IgM cross-reactivity could also be seen at lower levels between TBEV and WNV [14] and between TBEV and JEV [49]. However, no occurrence of these viruses has been reported in Belgium so far, but some participants in this study visited one or more countries within the prior three-month period.

Among the IgG ELISA- and IFA-positive students, six had a history of yellow fever vaccination. Three of these cases were confirmed through epidemiological investigation (one of them also has a history of symptoms of dengue), while the remaining three could not be contacted but were very likely vaccinated, as yellow fever vaccination is mandatory in their country of origin. For another student, we were not able to receive other epidemiological information. This observation is in line with the literature. In fact, when testing the yellow fever- and dengue virus-positive specimens, problems with Orthoflavivirus cross-reactivity were observed using commercial TBE ELISA tests (e.g., [43,48,50]). In the study of Litzba et al. [43], an IFA was also positive but at lower level for sera from patients infected with dengue and from yellow fever-vaccinated people.

TBEV-specific IgG antibodies were detected and confirmed by a virus neutralization test (VNT) in 0.5% (3/556; 95% CI, 0.1–1.6%) of nature management workers in the Netherlands [51]. This percentage is similar to the percentage of 0.48% observed in our study. Another French serological survey (IgG ELISA) of field professionals who have frequent and regular contact with the forest environment was conducted in the regions of Alsace, Lorraine, Franche-Comté, Champagne-Ardenne, and Bourgogne [52]. In this survey, an apparent prevalence of 3.4% was found, a result relatively close to that in our study (4,8%). In Germany, a percentage of 3.4% was also found for anti-TBE-virus IgG, close to the French result [53]. However, caution is needed because difference in prevalence between countries is difficult to assess properly due to difference in sampling design, groups of people tested, testing methodology, and time frames.

Several studies failed to find the virus or its genome in ticks (*I. ricinus*) collected through flagging in the surrounding nature of Belgian autochthonous cases and in known hotspots across Belgium between 2018 and 2024 despite the high quantities of ticks (N = 4590) analyzed [54]. However, very recently, the virus was detected, for the first time, in ticks collected in the province of Liège [35], suggesting the possibility of detecting autochthonous cases (time/space similarity).

One previous survey was performed in northern Belgium on forestry workers exposed to tick bites during professional activities and all results were seronegative [34]. However, there is now evidence [55] that TBE virus is introduced a long time before the first human cases are detected. Therefore, missing human antibodies may be no evidence of non-existence of TBE virus.

Considering the incidence of tick bites reported by citizen science [56] and the distribution of forested areas in Belgium, i.e., 700,000 hectares, with 79.8% in Wallonia (southern Belgium), 19.9% in the Flemish Region (northern Belgium), and 0.3% in the Brussels-Capital Region [57], the risk of tick bites is likely higher in southern Belgium. In northern France, using ELISA IgG tests supported by serum neutralization tests, TBEV seroprevalence was estimated as 0.14% (95% CI: 0.05–0.42) in forestry workers (participants with a history of TBEV vaccination were excluded) [58]. Therefore, we recommend conducting a similar serological survey, coupled with epidemiological investigation, among forestry workers from southern Belgium.

Students from the University of Liège are distributed across four campuses: in the city center of Liège and in Sart Tilman (province of Liège), in Gembloux (province of Namur), and in Arlon (province of Luxembourg). The Sart Tilman campus, which lies over several hundred hectares of green spaces and forest, hosts seven of the university’s eleven faculties. Gembloux Agro-Bio Tech, the faculty of bioengineering, covers 130 hectares, including 17 hectares of woodland. The Arlon campus is exclusively dedicated to the environment and is situated in a 3-hectare green setting. On these three campuses, forested and green areas are frequently used for recreational and academic activities, with possible contacts with ticks. Given that southern Belgium is a region with a significant increasing risk of human TBE infection [39] and due to the recent first virus detection in ticks collected in the province of Liege [35], awareness is needed in student populations, such as that of the University of Liège. We recommend presenting the results of this survey and other studies (especially [35,39]) to the department of Hygiene and Health Protection at Work (SUPHT) at the University of Liège to initiate discussions on potential actions specific to the student population to mitigate TBE risk. In terms of education, several initiatives should be encouraged from a One Health perspective, including all faculties and disciplines (medicine, veterinary medicine, agronomy, sociology, environment, ecology, economy, architecture, etc.). In the short term, the development of several topics for master’s and doctoral theses dedicated to ticks and tick-borne diseases in the environment of the university should be promoted. Some examples could be surveys focusing on forestry and recreational areas used by students on the campuses, estimation of tick bites through surveys and citizen science, development of video games to raise awareness about tick-related risks, or the development of mobile applications to manage the risk based on the locations on the campuses and the results of previous surveys. These proposals should be the motivation to develop practical exercises integrating students’ activities and new lines of research within university laboratories. Consequently, students from live sciences faculties (particularly future medical doctors, veterinarians, bioengineers) will be more aware of ticks and tick-borne diseases.

Until now, few human cases of TBE were observed in Belgium. Indeed, the country is classified as an affected country for TBE although the risk of infection is currently quite low. From 2013 onwards, 32 TBE cases were reported including 2 possibly autochthonous cases in 2018, 3 autochthonous cases in 2020, and 2 autochthonous and a 3rd possibly autochthonous case in 2024 [10]. These numbers are likely underestimated due to under-diagnosis and/or underreporting [23].

Several studies carried out on different populations of domestic animals (cattle, sheep, dogs) and wild animals (deer, roe deer, and wild boar) in Belgium report wide animal exposure to TBEV [25,26,27,28,29,33]. Regarding wild boars, on which several studies are available in northern Belgium, and despite some design differences between studies, an increasing trend in seroprevalence was observed from around 4.2% in 2013 up to 9.27% in 2020 [28,29]. This observation can be related to the results of a multi-scale modeling from Dagostin et al., which found a significant increase in human TBE infection risk in western Germany, southern Belgium, eastern France, and Switzerland [39]. Drivers of increasing TBE cases were recently listed, with changes in human behavior cited as the most important factor [36]. This factor was also proposed as a plausible explanation for the first autochthonous TBE cases reported in 2020 [32]. Further studies on behavioral and activity changes are needed in Belgium to refine awareness campaigns targeting populations at risk. Moreover, in epidemiological investigation, recent activities such as forest sightseeing and hiking may be included.

## 5. Conclusions

Exposure was assessed for the first time, but we did not find proof of natural TBEV exposure. This finding contributes to better decision-making in public health and prevention and management of tick-borne diseases in the context of climate change. Awareness among all students should be prioritized, with prevention measures against tick bites, particularly during forest and recreational activities contributing to risk.

## Figures and Tables

**Table 1:** Participants tested by ELISA (IgG or IgM) as a function of the faculty of origin.

Faculty	Tested Positive	Tested Negative	Total
Architecture	0	5	5
Law, Political Science and Criminology	0	2	2
Gembloux Agro-Bio Tech	0	3	3
HEC Liège-School of Management	1	11	12
Medicine	0	2	2
Veterinary Medicine	3	108	111
Philosophy and Letters	1	21	22
Psychology, Speech Therapy and Education Sciences	0	2	2
Sciences	0	2	2
Applied Sciences (Engineering)	4	27	31
Social Sciences	2	11	13
Other	0	2	2
Total	11	196	207

**Table 2:** Tick-borne encephalitis serological results and summary of epidemiological investigation.

ID	Faculty	Ratio ELISA IgM	Result ELISA IgM	Result IFAT IgM	Ratio ELISA IgG	Result ELISA IgG	Result IgG IFAT	TBE PRNT 50	TBE PRNT 90	Sex	Age (Years)	Nationality	Symptom History	Country Visited (alpha-2 code)	Vaccination
4	Philosophy and Letters	0.11	N	-	1.13	P	N	-	-	F	25	BE	No	HR, KH, SE	FSME-Immun 2 doses in 2014
34	Applied sciences	0.09	N	-	3.32	P	P (1/100)	<10	<10	M	30	BE	No	Europe, Latin America	Yellow Fever
89	Applied sciences	0.12	N	-	2.90	P	P (1/100)	<10	<10	M	34	CM	N.D.A.	N.D.A.	N.D.A. ^a^
93	School of Management	0.15	N	-	5.20	P	P (1/1000)	<10	<10	M	27	CM	N.D.A.	N.D.A.	N.D.A. ^a^
95	Applied sciences	0.16	N	-	5.51	P	P (≥1/1000)	<10	<10	M	32	BE	Dengue	CO, SE	Yellow Fever
105	Applied sciences	0.24	N	-	3.12	P	p (1/100)	226	106	F	29	DE	N.D.A.	N.D.A.	N.D.A.
121	Social sciences	0.12	N	-	1.72	P	N	-	-	F	46	BE	No	DE, Scandinavia	No
122	Social sciences	0.17	N	-	2.37	P	P (1/100)	<10	<10	F	34	CM	N.D.A.	N.D.A.	N.D.A. ^a^
227	Veterinary medicine	3.53	P	N	0.06	N	-	-	-	F	25	BE	No	ZA, JP, MA, SP	No
228	Veterinary medicine	0.10	N	-	2.92	P	P (1/10)	<10	<10	F	26	BE	No	CM, FR	Yellow Fever
230	Veterinary medicine	0.06	N	-	1.81	P	P (1/10)	<10	<10	F	25	FR	No	ZA	No

Legend: P, positive; N, negative; BE, Belgium; KH, Cambodia; CM, Cameroon; CO, Colombia; DE, Germany; FR, France; JP, Japan; MA, Morocco; SP, Spain; SE, Sweden; ZA, South Africa; N.D.A., no data available; FSME-Immun, inactivated TBE vaccine; ^a^, yellow fever vaccination is mandatory in Cameroon (https://www.wanda.be/en/landen/cameroon/ (15 August 2025)).

## Data Availability

Data is contained within this article.
